# Quantum chemical modeling of the reaction path of chorismate mutase based on the experimental substrate/product complex

**DOI:** 10.1002/2211-5463.12224

**Published:** 2017-05-02

**Authors:** Daniel Burschowsky, Ute Krengel, Einar Uggerud, David Balcells

**Affiliations:** ^1^ Department of ChemistryUniversity of OsloNorway; ^2^ Present address: Leicester Institute of Structural and Chemical BiologyUniversity of LeicesterLeicesterUK

**Keywords:** chorismate mutase, Claisen rearrangement, enzyme catalysis, pericyclic reaction, transition state stabilization

## Abstract

Chorismate mutase is a well‐known model enzyme, catalyzing the Claisen rearrangement of chorismate to prephenate. Recent high‐resolution crystal structures along the reaction coordinate of this enzyme enabled computational analyses at unprecedented detail. Using quantum chemical simulations, we investigated how the catalytic reaction mechanism is affected by electrostatic and hydrogen‐bond interactions. Our calculations showed that the transition state (TS) was mainly stabilized electrostatically, with Arg90 playing the leading role. The effect was augmented by selective hydrogen‐bond formation to the TS in the wild‐type enzyme, facilitated by a small‐scale local induced fit. We further identified a previously underappreciated water molecule, which separates the negative charges during the reaction. The analysis includes the wild‐type enzyme and a non‐natural enzyme variant, where the catalytic arginine was replaced with an isosteric citrulline residue.

**Abbreviations**
APT: atomic polar tensor
BsCM: *Bacillus subtilis* chorismate mutase
CM: chorismate mutase
DFT: density functional theory
IRC: intrinsic reaction coordinate
PCM: polarizable continuum model
QM/MM: quantum mechanics/molecular mechanics
TSA: transition state analog
TS: transition state

Chorismate mutases (CMs) are enzymes that catalyze the pericyclic rearrangement of chorismate (**1**) to prephenate (**2**). The reaction progresses via a chair‐like transition state (TS) (**1**→**2**) 1 (Fig. 1) and is formally a Claisen rearrangement. This unimolecular reaction occurs readily both in solution and by enzyme catalysis 2, 3, 4, representing a tractable model system in many ways. In particular, the CM of *Bacillus subtilis* (BsCM) has been extensively studied biochemically and computationally 1, 4, 5, 6, 7, 8, 9, 10, 11. With only very few enzymes catalyzing a pericyclic reaction 12 (and references therein) 13, 14, 15, CM has become the benchmark in computational chemistry. Particularly intriguing is the fact that the enzyme is not involved directly in the actual chemical transformation, but nevertheless accelerates the reaction 2‐million‐fold over the spontaneous reaction in water 5.

**Figure 1:**
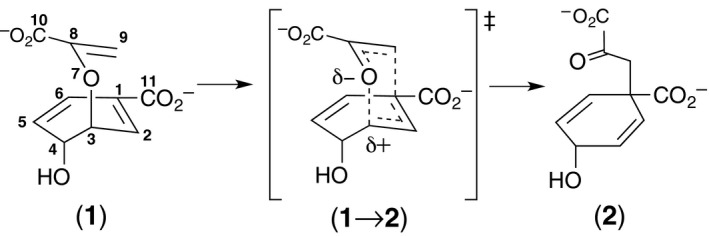
Claisen rearrangement from chorismate (**1**) to prephenate (**2**) via a chair‐like TS (**1**→**2**).

A milestone in the elucidation of the enzyme reaction mechanism was reached when the crystal structure of CM was solved by Lipscomb and coworkers in 1993, and refined to 2.2 Å resolution 7, 8. The active site structure in complex with Bartlett's TS analog (TSA) 16 and reaction product (**2**) strongly suggested that the enzymatic mechanism proceeds as a pericyclic process, similar to the uncatalyzed reaction. The structure provided the basis for the first quantum and molecular mechanics (QM/MM) study on CM, which suggested that substrate conversion may involve a combination of substrate strain and TS stabilization 17. Arg90 was found to play a central role, as confirmed by site‐directed mutagenesis 10, 11, 18, 19. Subsequent computational studies explored the potential reaction path, including chorismate pre‐equilibration, and its activation energy 9, 20, 21, 22, 23, 24, 25, 26, 27, 28, 29, 30, 31, 32 as well as the role of individual active site residues 25, 33 and solvation 21, 24, 25, 26, 32, 34. For over a decade, there was a heated debate about the question if TS stabilization or ground state destabilization provides the main driving force for the rate enhancement 11, 22, 31, 35, 36. A series of high‐resolution structures of a non‐natural CM variant (Arg90Cit) in complexes with substrate product and TS analog, recently provided strong experimental evidence for electrostatic TS stabilization, in line with Pauling's paradigm 37.

High‐resolution crystal structures elucidate structural details at exquisite precision; however, they usually only provide structural snapshots, and do not reveal entire reaction paths. These can be obtained from computational simulations, but are only meaningful if based on accurate 3D structures. It is well known that already small perturbations such as the rotation of a hydroxyl group in chorismate 38 can significantly influence molecular simulations. Full exploration of an enzymatic reaction therefore requires a combination of experimental and computational approaches. The large variation in computational models of the CM Arg90Cit active site 22, 27, 28, 39 suggests that the structural basis for accurate CM simulations has so far been limited. The recent high‐resolution crystal structures of wild‐type and Arg90Cit CM 40 provided an ideal basis for improved simulations. This is true in particular for the elusive substrate complex of BsCM, which was recently trapped and refined to a resolution of 1.7 Å 40.

Given the choice between different modeling methods, we deliberately chose a QM‐only method over QM/MM simulations. The hybrid density functional theory (DFT) method with B3LYP exchange‐correlation functional 41 is known to be a very useful electronic structure method for treating large structures. It allows accurate simulations at low costs, as long as the input data are of high quality and appropriate validations are ensured, e.g. by testing a range of dielectric constants 29, 42, 43. Furthermore, it minimizes the risk of having the energy minimization stuck in local energy minima, which are common in MM simulations.

Here, we present quantum chemical simulations at the DFT (B3LYP) level on a cluster model 42, 43, 44, 45, 46, 47 of BsCM. The aim of the study was to explore the reaction path of this model enzyme based on the recent high‐resolution structural complexes.

## Methods

**Figure 2:**
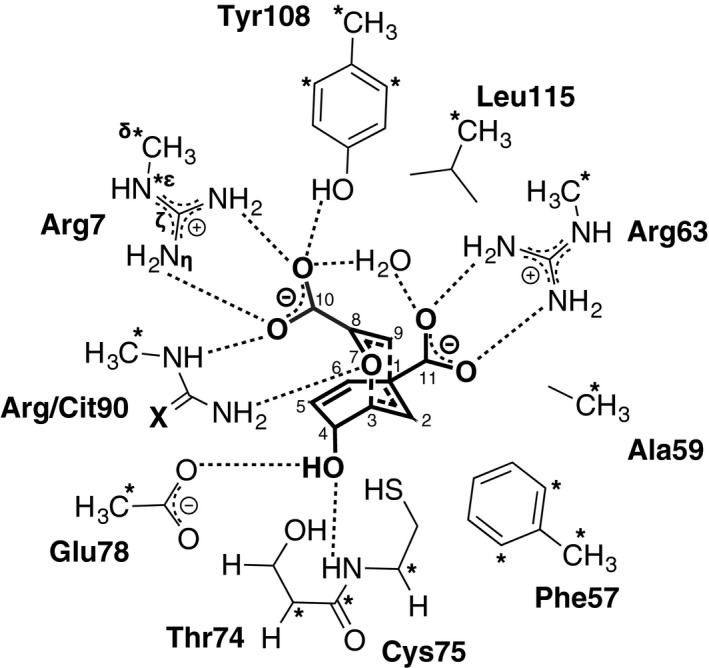
Schematic representation of the active site model of BsCM containing the TS (**1**→**2**) that was used for geometry optimization. X = O for Cit90 and X = ${\text{NH}}_{2}^{+}$ for Arg90. Asterisks indicate atomic coordinates that were frozen during all optimization steps. Hydrogen bonds to the TS are indicated with dashed lines.

All geometries presented in this study were optimized using Gaussian09 48 at the DFT B3LYP/6‐31G(d) level (see Table S1 and supplementary coordinates). For the models, we selected the residues comprising the active site of BsCM Arg90Cit of the substrate/product‐bound structure (PDB‐ID 3ZP7 40), in accordance with the methodology described by Sevastik and Himo 49. nciplot 2.0 50, 51 was used to visualize the nonbonding interactions of all residues, which allowed removal of noncontributing atoms (e.g. protein backbone and noninteracting methyl groups) and to further decrease the size of the system. All atomic positions were optimized except those noted in Fig. 2, which were kept frozen to preserve the essential structural features observed in the crystal structure and to prevent the side chains from drifting into positions that would be occupied by the surrounding residues. For residue 90, we used either an oxygen atom or an ${\text{NH}}_{2}^{+}$ group for citrulline and arginine, respectively. After optimization of the chorismate‐ and prephenate‐bound states, TS starting geometries were inferred from the substrate‐bound end states, and their geometries were subsequently optimized. The influence of the environment was explored by refining the energies (*E*) with single‐point calculations on the optimized geometries with the 6‐311+G(d,p) basis set and used the thermochemical corrections from the geometry optimizations to calculate the free energies (*G*); i.e. *G* = *E* + (*G*_opt_ − *E*_opt_). The single‐point calculations were repeated using the polarizable continuum model (PCM) method for dielectric constants ε ranging from 1 to 40 (see Table S1). After geometry optimization of the chorismate‐ and prephenate‐bound states, approximately 10 imaginary frequencies were found due to the freezing of several atoms. Subsequently, initial transition state geometries were guessed from the chorismate‐bound state and optimized at the B3LYP/6‐31G(d) level of theory. TS showed a single additional imaginary frequency involving the vibration of chorismate along the reaction pathway. These optimized coordinates were then used for single‐point calculations at the B3LYP/6‐311+G(d,p) level of theory, with thermochemical corrections and PCM calculations being performed as described above.

The TS geometries were used as starting points to follow the intrinsic reaction coordinate (IRC) toward the substrate and product sides of the energy barrier. A first run with a step size of only 0.001 Bohr was used to relax rotations of terminating frozen methyl groups, which would otherwise induce an offset from the TS to the residual data points. The output was used in a second run with the standard step size of 0.01 Bohr in order to follow the IRC. The endpoints (substrate‐ and product‐bound active sites) that were obtained from the IRC calculation were then subjected to geometry optimization (B3LYP/6‐31G(d)) for comparison with the originally geometry‐optimized active sites from the crystal structures (Fig. S2).

The effect of introducing empirical dispersion in the single‐point calculations for dielectric constants of 1 and 20 was investigated for validation purposes with the Grimme model (GD3) 52. Dispersion did not seem to have a major effect; it only changed the ∆∆*G* ^‡^ by 1 kJ·mol^−1^. In addition, nciplot 2.0 50, 51 was used to assess the presence of disruptive interactions, such as too close proximity between atoms or between charges of the same polarity. No such interactions were found in the model. Superimpositions and graphical representations of the active site coordinates were generated by using pymol 53.

The choice of the cluster‐PCM approach used in this work was based on previous studies of enzymatic reactions and other chemical systems involving both neutral and charged species 54, 55, including anions 56, 57, 58, even though it has the limitation of not exploring the large conformational space of the full system and its contribution to the Gibbs energy. However, given our prior knowledge of the rigidity of the system, we decided that this was not necessary. The electrostatic interactions were therefore described with an accurate quantum mechanical method based on DFT. The single negative charge in the Arg90Cit system was stabilized by including the interactions between the reactive center and seven different residues, including Arg7, Arg63, Thr74, Cys75, Glu78, Arg/Cit90 and Tyr108, which all form H‐bonds. The free energies given in the text include both the solvation effects and the thermochemistry (zero‐point, thermal and entropy energies).

## Results and Discussion

A quantum chemical model of the BsCM active site was prepared based on the substrate complex of the BsCM Arg90Cit variant (PDB‐ID 3ZP7 40). QM simulations were performed at the DFT (B3LYP) level in analogy to a study on 4‐oxalocrotonate tautomerase 49. Also in that case, citrulline was used as a neutral probe to replace a catalytic arginine and it was shown that concentrating on a quantum chemical model was sufficient for this enzymatic reaction, because it does not involve large conformational changes. When preparing the model, we identified a water molecule bridging the two carboxylate groups of the substrate/product molecules in several active sites in the crystal structure, a structural feature that was also observed in structurally unrelated CMs 59, 60, 61. This molecule's contribution to the hydrogen‐bonding network of the active site may be important for the energetics of the active site of BsCM by helping to stabilize the substrate in the active conformation. To our knowledge, this water molecule has not been included in previous computational simulations, presumably because it was not modeled in the original BsCM structures (PDB‐IDs 2CHT, 1COM) 7, 8. It is, however, present in all recent high‐resolution CM structures, regardless of origin or crystal form (e.g. PDB‐IDs 2W1A 61, 2FP2 60, 1ECM 59).

**Figure 3:**
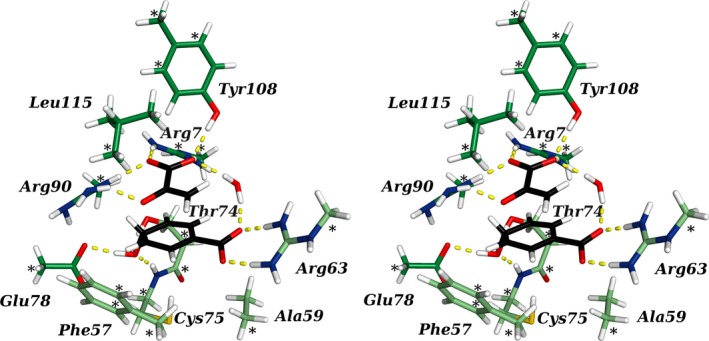
Stereo view of the transition state model of wild‐type BsCM. Asterisks denote the atomic coordinates that were frozen during optimization. The TS is depicted with black carbons, enzyme side chain carbons are shown in green, with light green denoting side chains from another subunit. Hydrogen bonds to the TS are shown in yellow.

For both the wild‐type and Arg90Cit variants of BsCM, a TS that was consistent with the pericyclic nature of the reaction was found (Fig. 3). Table 1 lists the interatomic distances of the bonds broken and formed (C3–O7 and C9–C1, respectively), the bond lengths of the chair‐like pericyclic TS as well as partial charges that were calculated using the atomic polar tensor (APT) model 62. These data show that there are no major differences in the geometry or charge distribution of the TS for either enzyme variant, i.e. the reaction most likely follows the same reaction pathway in both cases.

**Table 1:** TS atomic distances and APT charges

	Wild‐type BsCM	BsCM Arg90Cit
Atomic distance [Å]		
C1–C2	1.39	1.39
C2–C3	1.39	1.40
C3–O7	2.19	2.16
O7–C8	1.30	1.30
C8–C9	1.37	1.37
C9–C1	2.81	2.77
APT charge^a^		
C1	0.357	0.265
C2	−0.278	−0.217
C3	0.337	0.293
O7	−0.585	−0.515
C8	0.150	0.178
C9	−0.182	−0.221
C10O_2_ b	−1.043	−1.040
C11O_2_ b	−0.947	−0.956
C4	0.336	0.353
O4	−0.322	−0.282
C5	0.084	0.087
C6	−0.108	−0.116

^a^ APT charges with the contributions of hydrogens summed into heavy atoms. ^b^Charges averaged for the carboxylate atoms.

In order to confirm the nature of these TSs, we calculated the IRC 63, 64 following the reaction backward and forward from their optimized geometries (Fig. S1). The potential energy barrier of wild‐type BsCM was reduced by 22.6 kJ·mol^−1^ compared to BsCM Arg90Cit. This energy difference is amplified by 14.0 kJ·mol^−1^ upon introduction of the thermochemical corrections, which include the zero‐point, thermal and entropy energies; i.e., ΔΔ*G* ^†^ = 36.6 kJ·mol^−1^. This is in good agreement with the experimentally observed 2 × 10^4^‐fold decrease in *k*_cat_ when Arg90 is replaced by Cit, which corresponds to a ΔΔ*G* ^†^ = 27.2 kJ·mol^−1^ 11. In addition, the optimization of the endpoints that were obtained from the IRC calculations yielded almost identical geometries as optimizing the chorismate‐ and prephenate‐bound crystal structures, further supporting the nature of the TS and the robustness of the model used (see Fig. S2).

**Figure 4:**
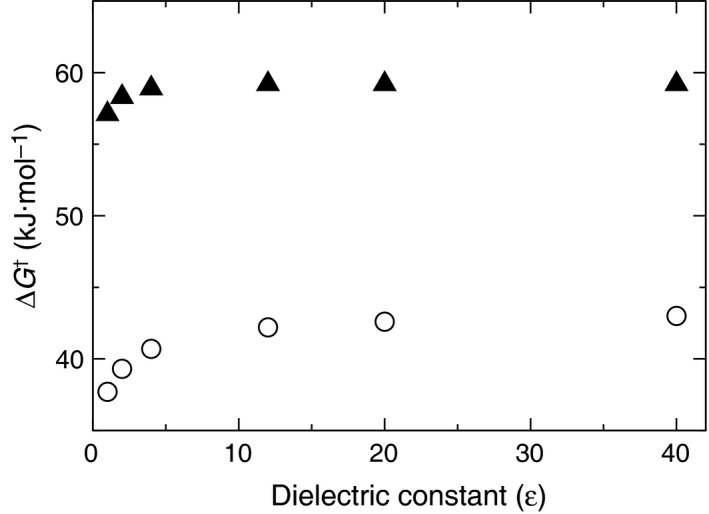
The energy barrier ∆*G* ^†^ of the chorismate‐to‐prephenate reaction, calculated at different dielectric constants ε for wild‐type BsCM (○) and BsCM Arg90Cit (▲). Convergence is reached from ε = 10. The corresponding energies are listed in Table S1).

We then tested several dielectric constant ε values with the PCM to account for the enzyme environment (for the relevant energies, see Table S1). The energy barriers increased for both the wild‐type and Arg90Cit variants, until convergence was reached from ε = 10 (Fig. 4), as expected for a robust system. This suggests that, upon increasing the polarity of the environment, the substrate‐bound structure is more stabilized than the TS complex due to a larger charge delocalization in the latter. Once converged, the difference between the energy barriers of each variant is approximately 18 kJ·mol^−1^, which is also in reasonable agreement with the experimental estimate of 27.2 kJ·mol^−1^. The decrease of ΔΔ*G* ^†^ upon increasing the dielectric constant is consistent with the hypothesis that electrostatic effects play the major role.

**Figure 5:**
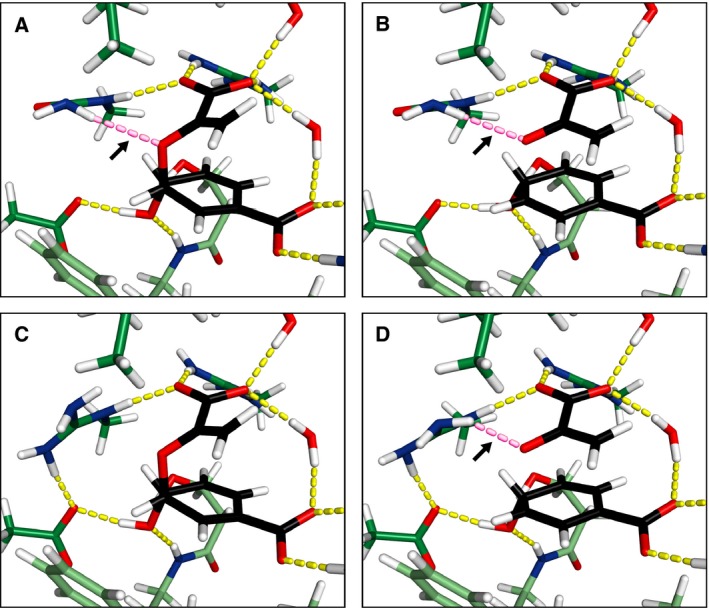
Close‐up view of the active site model after energy optimization. BsCM Arg90Cit with chorismate (A) and the TS (B) in the active site is contrasted with wild‐type BsCM, bound to chorismate (C) and the TS (D). The coloring is equivalent to Fig. 3, except for the hydrogen bond between residue 90 and the substrate/TS, which is colored in magenta and marked with an arrow.

Upon close inspection of the different structures, we identified an interesting feature of the active site (Fig. 5, Table 2). In the Arg90Cit variant, which we used as a starting structure, the distal NHη of the Cit90 side chain forms a hydrogen bond with the ether oxygen O7 of chorismate (Figs 2 and 5A). When the TS was reached, this hydrogen bond was still in place in our simulations (Fig. 5B). In contrast, the wild‐type residue Arg90 does not engage in such a hydrogen bond with the substrate. Instead the plane formed by its guanidinium group is tilted by 29° with respect to the urea group of BsCM Arg90Cit to form a salt bridge with Glu78. This places Arg90 at an angle that is unfavorable for hydrogen‐bond formation to O7 of chorismate (Fig. 5A,C). When approaching the TS, however, the bond that is to be broken was elongated to 2.2 Å (Table 1) and placed the ether oxygen with the developing negative partial charge in close proximity to Arg90. The positively charged guanidinium group of Arg90 then tilted by 13° toward O7 (Fig. 5C,D), selectively facilitating the stabilization of the TS by the generation of a hydrogen bond, additionally contributing to the lower energy barrier observed for wild‐type BsCM.

**Table 2:** Interatomic distances, in Å, relevant to hydrogen bonding between residue 90 and the substrate‐ and TS‐bound structures

	Wild‐type BsCM	BsCM Arg90Cit
Res90 to substrate		
NHε–$C10O_{2}^{-}$	1.75	1.93
NHη–O7	2.84^a^	2.40
Res90 to TS		
NHε–$C10O_{2}^{-}$	1.79	1.95
NHη–O7	1.91	2.17

a Geometrically not favorable for hydrogen‐bond formation.

As an interesting aside, when comparing the interaction with Arg90 in the experimental TSA complex structure 8 with the DFT‐optimized geometries, the former is seemingly closer to the substrate‐ than to the TS‐bound geometry. However, upon carefully superimposing the active sites by ignoring the flexible parts and hydrogens (Fig. S3), the ether oxygen of the TSA is almost perfectly placed for the crucial interactions. The TSA structure is thus a good starting point for future inhibitor design.

## Conclusion

In summary, we have isolated and characterized the electrostatic effects in CM catalysis. This type of analysis is not trivial from a purely energetic point of view, because the charges not only interact within the substrate, but also with the structural framework of the active site. Nevertheless, by probing the reaction with both DFT geometry optimizations and single‐point energy calculations for different dielectric constants, we closely approached the experimentally derived energies, confirming Arg90 as the key contributor to electrostatic TS stabilization. In addition, we showed how this is achieved, namely by selective hydrogen‐bond formation to the ether oxygen O7 in the TS, facilitated by a localized induced fit. We further identified a conserved water molecule that has previously been neglected in computational studies, and whose contribution to the Claisen rearrangement of chorismate to prephenate would be interesting to further explore. However, explicitly assessing the molecule's potential influence on the dynamics of the system was beyond the scope of the present manuscript.

Clearly, we have come a long way from the early days of minimalistic modeling of the CM reaction 5, 9, 17, 65 to the current elucidation of the enzyme mechanism at unprecedented detail.

## Data Accessibility

Research data pertaining to this article is located at figshare.com: https://dx.doi.org/10.6084/m9.figshare.5001902 [Correction added after online publication on 17 May 2017: Data Accessibility section added].

## Author contributions

UK conceived the study. EU and DBa designed the study. DBu performed the calculations, analyzed the results and drafted the paper. DBu, UK, EU and Dba wrote the paper.

## Supporting information


**Fig. S1.** The intrinsic reaction coordinate [1,2] for the chorismate‐to‐prephenate reaction, starting with the transition states of Arg90 (open circles) and Cit90 (closed triangles).
**Fig. S2.** Superimpositions of the geometry‐optimized active sites (green carbons) with geometry‐optimized structures of the IRC calculations’ endpoints (purple carbons).
**Fig. S3.** Stereo view of the superimposition of the TS in the geometry‐optimized active site of wild‐type BsCM with the corresponding TSA‐bound structure (active site between chains B/A, PDB‐ID: 2CHT [3]).
**Table S1.** Energies (kJ·mol^−1^) of active site geometry optimizations and single‐point calculations for different dielectrical constants ε.
