# Supplementary material for: Quantum chemical modeling of the reaction path of chorismate mutase based on the experimental substrate/product complex
Source: FEBS Open Bio. 2017 May 2;7(6):789–97. doi: 10.1002/2211-5463.12224 (PMC5458464; doi:10.1002/2211-5463.12224)
Supplement: Supplementary file 1 — Fig. S1. The intrinsic reaction coordinate [1,2] for the chorismate‐to‐prephenate reaction, starting with the transition states of Arg90 (open circles) and Cit90 (closed triangles). Fig. S2. Superimpositions of the geometry‐optimized active sites (green carbons) with geometry‐optimized structures of the IRC calculations’ endpoints (purple carbons). Fig. S3. Stereo view of the superimposition of the TS in the geometry‐optimized active site of wild‐type BsCM with the corresponding TSA‐bound structure (active site between chains B/A, PDB‐ID: 2CHT [3]). Table S1. Energies (kJ·mol−1) of active site geometry optimizations and single‐point calculations for different dielectrical constants ε. [file FEB4-7-789-s001.docx]

Quantum chemical modeling of the reaction path of chorismate mutase based on the experimental substrate/product complex

Daniel Burschowsky^1,2,*^, Ute Krengel^1^, Einar Uggerud^1^, David Balcells^1,*^

^1^ Department of Chemistry, University of Oslo, Oslo, Norway

^2^ Present address: Leicester Institute of Structural and Chemical Biology, University of Leicester, Leicester, UK

^*^ Corresponding authors: db379@le.ac.uk, david.balcells@kjemi.uio.no

SUPPORTING INFORMATION

**Supplementary Table**

**Table S1:** Energies (kJ·mol^-1^) of active site geometry optimizations and single-point calculations for different dielectrical constants *ε*.

|  | BsCM | geometry optimization | | PCM single-point energies **E** | | | | | |
| --- | --- | --- | --- | --- | --- | --- | --- | --- | --- |
|  |  | **E_opt_** | (**G_opt_** – **E_opt_**)*^a^* | *ε* = 1 | *ε* = 2 | *ε* = 4 | *ε* = 12 | *ε* = 20 | *ε* = 40 |
| chorismate | wild type | -9281710.6 | 2917.7 | -9284423.1 | -9284494.6 | -9284544.7 | -9284588.5 | -9284598.8 | -9284607.1 |
|  | Arg90Cit | -9332507.8 | 2829.4 | -9335268.1 | -9335406.7 | -9335493.9 | -9335564.3 | -9335580.2 | -9335592.7 |
| TS | wild type | -9281663.5 | 2906.7 | -9284374.4 | -9284444.3 | -9284493.1 | -9284535.3 | -9284545.2 | -9284553.1 |
|  | Arg90Cit | -9332458.4 | 2834.3 | -9335216.0 | -9335353.3 | -9335440.0 | -9335510.1 | -9335525.9 | -9335538.5 |
| prephenate | wild type | -9281794.7 | 2932.1 | -9284505.8 | -9284574.3 | -9284622.6 | -9284664.9 | -9284674.9 | -9284682.9 |
|  | Arg90Cit | -9332592.5 | 2855.7 | -9335341.7 | -9335471.2 | -9335554.3 | -9335622.7 | -9335638.4 | -9335651.0 |

*^a^* (**G_opt_** – **E_opt_**) denotes the thermochemistry correction that was used to calculate free energies (**G**).

**Figure S1:** The intrinsic reaction coordinate [[1](#_ENREF_1), [2](#_ENREF_2)] for the chorismate-to-prephenate reaction, starting with the transition states of Arg90 (open circles) and Cit90 (closed triangles). ∆*E* is shown relative to the substrate-bound ground state of the active site and was not corrected for thermal energy.

**Figure S2:** Superimpositions of the geometry-optimized active sites (green carbons) with geometry-optimized structures of the IRC calculations’ end points (purple carbons). The wild-type active sites show r.m.s.d. distances of all heavy atoms of 0.06 Å in the substrate-bound structure (**a**) and 0.10 Å in the product-bound structure (**b**), respectively, whereas the Arg90Cit variant active sites show 0.21 Å and 0.36 Å for substrate- (**c**) and product-bound (**d**) structures.


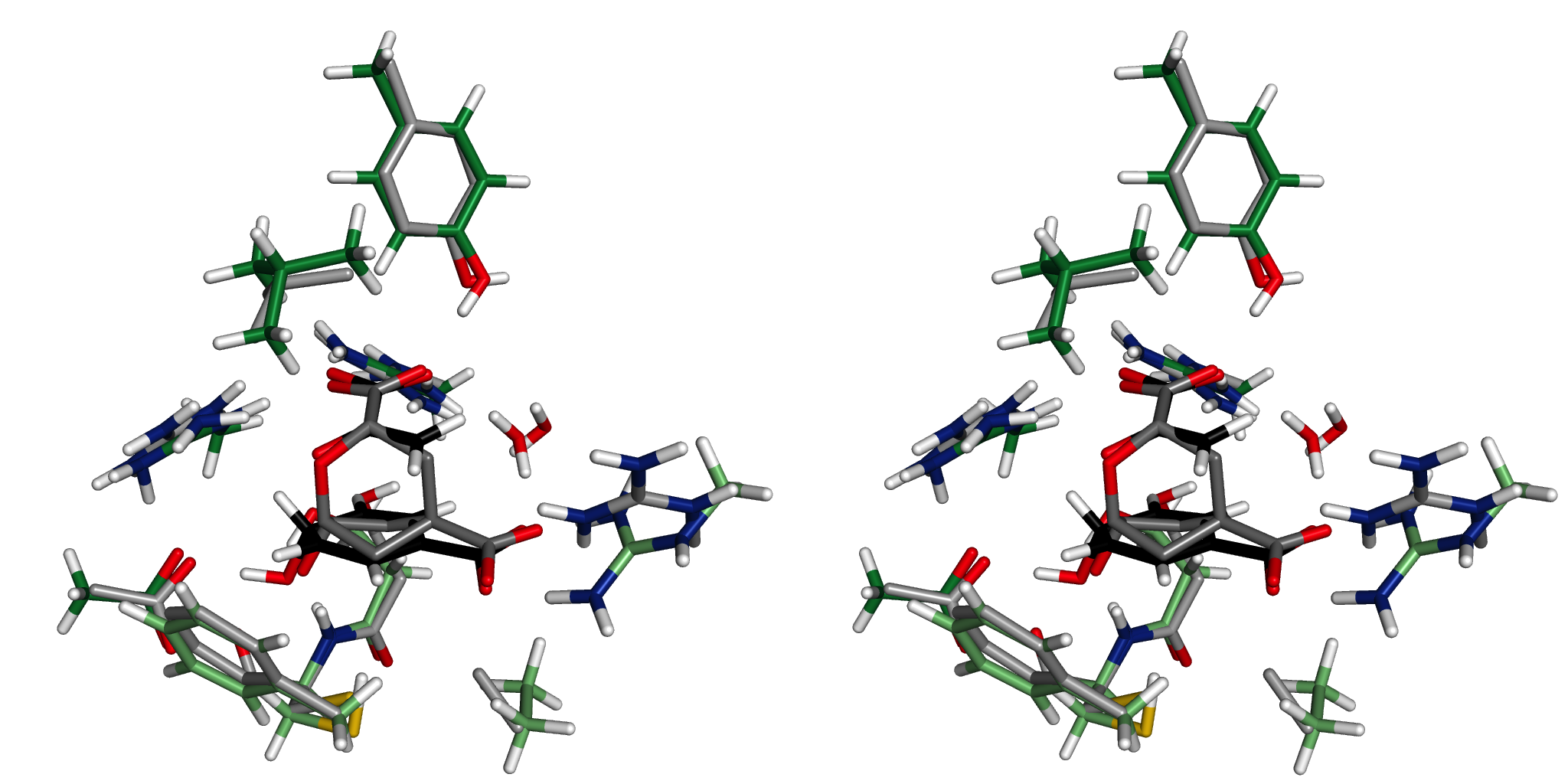


**Figure S3:** Stereo view of the superimposition of the TS in the geometry-optimized active site of wild-type BsCM with the corresponding TSA-bound structure (active site between chains B/A, PDB-ID: 2CHT [[3](#_ENREF_3)]). The superimposition was performed by aligning the C, O, N and S atoms of the non-flexible side chains (i.e. excluding Arg63).

**References**

1. Fukui, K. (1981) The path of chemical reactions - the IRC approach, *Acc Chem Res.* **14**, 363-368.

2. Hratchian, H. P. & Schlegel, H. B. (2005) Chapter 10 - Finding minima, transition states, and following reaction pathways on ab initio potential energy surfaces in *Theory and Applications of Computational Chemistry* (Dykstra, C. E., Frenking, G., Kim, K. S. & Scuseria, G. E., eds) pp. 195-249, Elsevier, Amsterdam.

3. Chook, Y. M., Gray, J. V., Ke, H. & Lipscomb, W. N. (1994) The monofunctional chorismate mutase from *Bacillus subtilis* - Structure determination of chorismate mutase and its complexes with a transistion state analog and prephenate, and implications for the mechanism of the enzymatic reaction, *J Mol Biol.* **240**, 476-500.
